# Addressing Hypertension Disparities via Systems Dynamics: Insights From Community Health Connections

**DOI:** 10.7759/cureus.68763

**Published:** 2024-09-06

**Authors:** Kavin Krishna, Mark Franciosa

**Affiliations:** 1 Health Policy, Stanford University, Redwood City, USA; 2 Internal Medicine, Community Health Connections, Fitchburg, USA

**Keywords:** access to healthcare, diversity and equity in medicine, health care disparities, hypertension, socio-economic factors, systems thinking

## Abstract

Background and objective

Hypertension remains a major public health challenge in the United States, disproportionately affecting various demographic groups. Significant disparities persist in hypertension prevalence and control due to interactions between socioeconomic factors, healthcare access, and systemic inequities. In this study, we aimed to determine the impact of socioeconomic and healthcare factors on hypertension control among patients at Community Health Connections (CHC), a Federally Qualified Health Center serving 36 communities in North Central Massachusetts, and identify effective interventions using systems dynamics modeling to promote health equity.

Materials and methods

This was a retrospective observational study using data from 2023 CHC patients, and the National Health and Nutrition Examination Survey (NHANES) 2011-2014. Systems dynamics modeling was employed to visualize interactions among factors influencing hypertension outcomes. The study included 4,870 CHC patients. Participants were selected based on clinical records and comprised 3,690 White participants (76%), 464 Black/African American participants (10%), 108 Asian participants (2%), and 608 classified as Other/Non-reported (12%). The cohort included 2,490 males (51%) and 2,380 females (49%). Socioeconomic factors (e.g., race, age, insurance status) and healthcare access were the study variables. The primary outcome was hypertension control, defined as a blood pressure reading of <140/90 mmHg. The study measured control rates across different demographic groups and assessed the impact of socioeconomic and healthcare factors on these rates.

Results

Among the 4,870 CHC patients, 3,007 (62%) achieved hypertension control. The overall hypertension control rate varied significantly by race: White: 68.6%, Black/African American: 61.6%, Asian: 63.2%, and Other/Non-reported: 65.6%. Insured patients had a control rate of 67.7%, compared to 37.5% for uninsured patients (p<0.001). Systems dynamics models illustrated how socioeconomic disparities and healthcare access issues amplify health inequities. Key interventions identified include, but are not limited to, multidisciplinary care teams, community health worker programs, and telehealth services.

Conclusions

Addressing hypertension disparities among CHC patients requires a systemic approach integrating socioeconomic, healthcare, and policy-related interventions. Systems dynamics modeling provides a framework for designing and implementing targeted interventions, enhancing health equity, and improving hypertension control outcomes in underserved populations. Further research is needed to test the effectiveness of these interventions before their broad implementation.

## Introduction

Hypertension is a significant public health issue, disproportionately affecting various demographic groups in the United States [1]. Despite numerous public health initiatives, substantial disparities persist in hypertension prevalence and control due to interactions between socioeconomic factors, healthcare access, and systemic inequities [2]. These disparities contribute to higher rates of uncontrolled hypertension among minority and low-income populations, exacerbating the burden of cardiovascular disease and associated health outcomes.

Community Health Connections (CHC), a Federally Qualified Health Center serving 36 communities in North Central Massachusetts, provides healthcare services to a diverse patient population. The patients served by CHC are representative of those disproportionately affected by hypertension-related health disparities. Understanding the factors influencing hypertension control in this population is crucial for developing effective interventions that promote health equity.

This study aims to determine the impact of socioeconomic and healthcare-related factors on hypertension control among patients at CHC. By employing systems dynamics modeling, this study seeks to visualize and analyze the complex relationships between these factors and identify effective interventions to promote health equity. This approach allows for a deeper understanding of the systemic issues contributing to hypertension disparities and offers a framework for designing targeted interventions that can be implemented in similar underserved populations.

## Materials and methods

Data sources

This study utilized data from two primary sources: clinical records from CHC, and the National Health and Nutrition Examination Survey (NHANES) 2011-2014 [1]. The CHC dataset included 4,870 patients with detailed demographic, socioeconomic, and healthcare access information, while the NHANES dataset provided national trends and disparities in hypertension prevalence and control.

Literature review

An extensive literature review was conducted to identify key variables and causal relationships relevant to hypertension control. This review used multiple databases, including MEDLINE, OpenAIRE, OAIster, and others such as the Complementary Index, Academic Search Premier, and the Directory of Open Access Journals. These databases were selected for their comprehensive coverage of biomedical and public health research, as well as their inclusion of interdisciplinary studies that address socioeconomic and healthcare access factors. The search terms included 'hypertension control,' 'healthcare access,' 'socioeconomic disparities,' 'systems dynamics,' and 'causal loop diagrams.' Studies were selected based on their relevance to the key variables and their potential contribution to constructing a conceptual systems dynamics model. The review focused on empirical studies, systematic reviews, and theoretical papers that provided insights into the interactions between the selected variables.

Systems dynamics modeling

The systems dynamics models developed in this study aimed to explore the complex relationships and feedback loops between key variables influencing hypertension control, including race, age, sex, insurance status, socioeconomic status, and healthcare access. These models were conceptual, based on established findings in the literature, and did not involve new data simulations. The modeling process included several steps:

Model Construction

The key variables identified through the literature review were mapped using causal loop diagrams (CLDs). The initial models were created using Vensim, a tool well-regarded for its ability to handle complex system dynamics models. As the study progressed, Insight Maker was also employed for its user-friendly interface and ability to produce legible diagrams that facilitated clear communication of the models. The CLDs highlighted key feedback loops, both reinforcing and balancing, that could influence hypertension outcomes. For example, reinforcing loops, such as the relationship between socioeconomic status and healthcare access, were modeled based on literature showing that higher socioeconomic status typically leads to better healthcare access, which in turn improves hypertension control outcomes [3,4].

Model Validation

The CLDs were validated by comparing them against well-documented findings in the literature. For instance, the correlation between socioeconomic status and hypertension control was supported by multiple studies [5,6]. The models were refined iteratively to ensure consistency with these established relationships, providing a robust conceptual framework for understanding the systemic issues related to hypertension control.

Application and Future Research

The primary objective of these models was to provide a conceptual framework that could guide the understanding of systemic disparities in hypertension control. While the models did not involve new simulations, they were designed to identify potential leverage points for intervention based on existing evidence. Future research could build on these models by incorporating quantitative simulations to explore specific intervention scenarios.

Statistical analysis

Chi-square tests were employed to assess associations between categorical variables (e.g., race, sex, insurance status) and hypertension control outcomes. These statistical analyses, including chi-square tests, were conducted using Python. The retrospective nature of the study and potential biases in data collection are acknowledged as limitations. The statistical results provided additional validation for the conceptual models by confirming relationships that were also reported in the literature.

## Results

Demographic characteristics

The study sample from CHC [7] consisted of 4870 patients. Their demographic characteristics are summarized in Table 1.

**Table 1:** Demographic characteristics

Variable	Categories	N	Percentage (%)
Race	White	3690	76%
	Black/African American	464	10%
	Asian	108	2%
	Other/Non-reported	608	12%
Sex	Male	2490	51%
	Female	2380	49%
Age (years)	<40	513	11%
	40-59	2092	43%
	60+	2265	47%
Insurance status	Insured	4838	99%
	Uninsured	32	1%
Hypertension control	Met	3007	62%
	Not met	1452	30%
	Excluded	411	8%

Hypertension control by demographics

The control rates varied significantly across different demographic groups (Table 2).

**Table 2:** Hypertension control rates by various demographics DF: degrees of freedom

Hypertension control						
Race	Met (%)	Not met (%)	Chi-square statistic	P-value	DF	Significance
White	68.6	31.4	8.536	0.003	1	Positive
Black/African American	61.6	38.4	7.053	0.008	1	Negative
Asian	63.2	36.8	0.698	0.403	1	Negative
Other/Non-reported	65.6	34.4	0.895	0.344	1	Negative
Sex	Met (%)	Not met (%)	Chi-square statistic	P-value	DF	Significance
Male	66.2	33.8	3.23	0.07	1	Negative
Female	68.2	31.8	3.23	0.07	1	Positive
Age group (years)	Met (%)	Not met (%)	Chi-square statistic	P-value	DF	Significance
<40	65.7	34.3	0.22	0.641	1	Negative
40-59	68	32	0.12	0.729	1	Positive
60+	67.2	32.8	0.02	0.897	1	Negative
Insurance status	Met (%)	Not met (%)	Chi-square statistic	P-value	DF	Significance
Insured	67.7	32.3	11.82	0.001	1	Positive
Uninsured	37.5	62.5	11.82	0.001	1	Negative

Comparison with NHANES data

The NHANES data from 2011-2014 corroborates the findings from CHC, highlighting persistent disparities in hypertension control (Table 3).

**Table 3:** Hypertension control rates among various demographics: NHANES data NHANES: National Health and Nutrition Examination Survey

Demographic group	Hypertension prevalence (%)	Controlled hypertension (%)
Total	29.0	53.0
Age 18-39 (years)	7.3	37.4
Age 40-59 (years)	32.2	57.2
Age 60+ (years)	64.9	52.5
Non-Hispanic White	28.0	55.7
Non-Hispanic Black	41.2	48.5
Non-Hispanic Asian	24.9	43.5
Hispanic	25.9	47.4

Socioeconomic and healthcare-related factors

**Figure 1:**
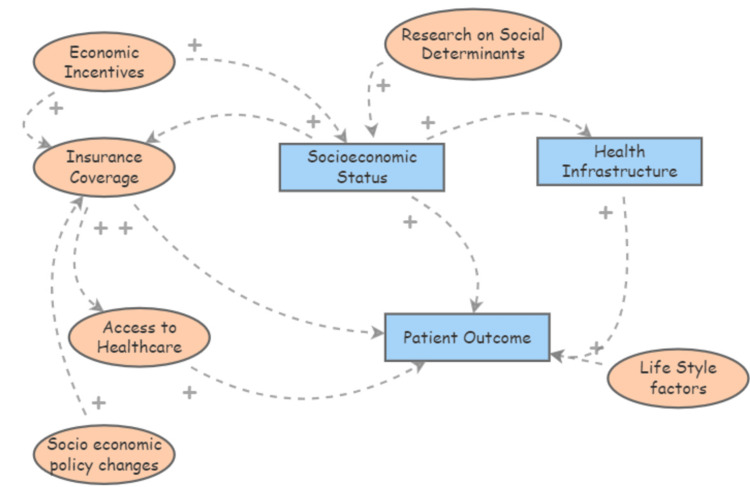
Causal loop diagram depicting how socioeconomic and healthcare-related factors affect hypertension control

**Figure 2:**
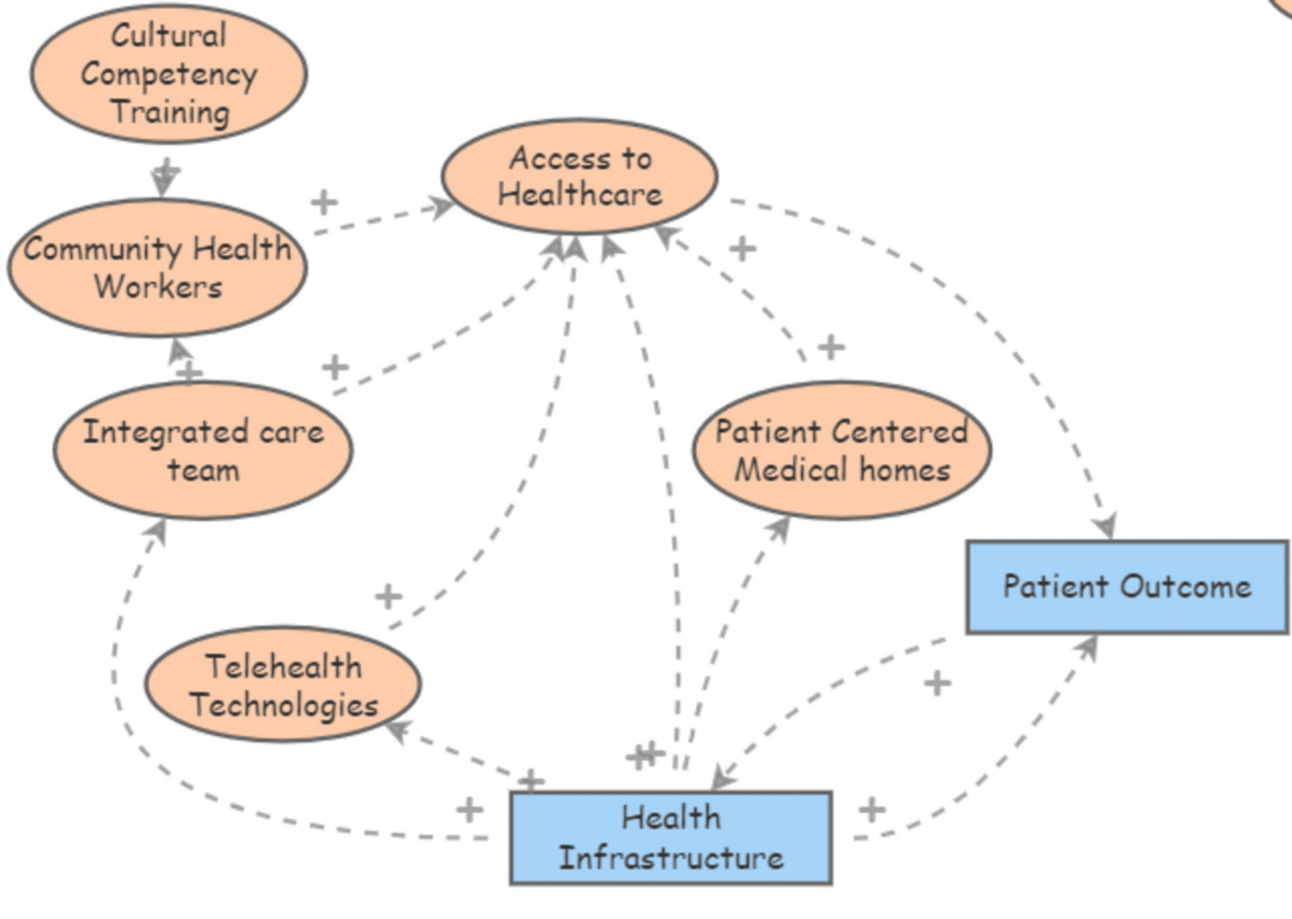
Causal loop diagram depicting how socioeconomic and healthcare-related factors affect hypertension control (Cont.)

**Figure 3:**
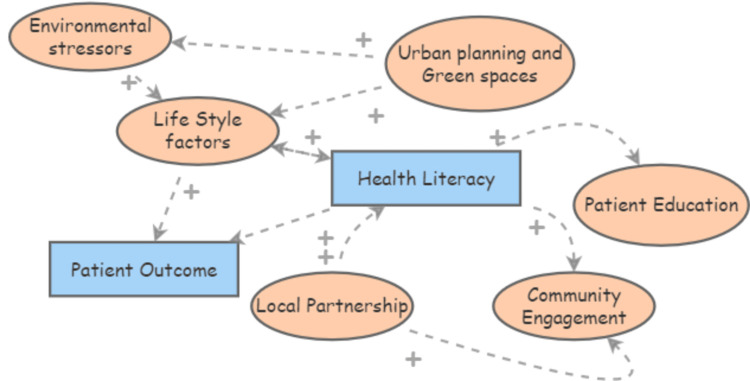
Causal loop diagram depicting how socioeconomic and healthcare-related factors affect hypertension control (Cont.)

Figures 1-3 illustrate how socioeconomic factors such as race, age, and insurance status interact with healthcare access and influence hypertension control. The relationship between socioeconomic factors and hypertension outcomes is well-documented in public health literature. Studies have shown that socially and economically disadvantaged groups, particularly racial and ethnic minorities, are at greater risk of adverse health outcomes due to systemic barriers to accessing quality healthcare [5,8]. For instance, research highlights how these social determinants interact with health inequities, leading to compounded disease burdens in minority communities, particularly during public health crises like the coronavirus disease 2019 (COVID-19) pandemic [9]. The examination of socioeconomic disparities in chronic disease outcomes demonstrates the significant impact of healthcare access and quality on hypertension control [3,10]. Also, policy changes enhancing access to healthcare can significantly improve outcomes for chronic conditions, as evidenced by the positive effects observed after expanding health insurance coverage [11]. Lastly, disparities in health infrastructure and lifestyle factors further compound the challenges in managing hypertension among disadvantaged groups [12,13].

Access to healthcare plays a crucial role in managing chronic conditions such as hypertension. Disparities in healthcare access, particularly in rural and underserved areas, exacerbate these challenges. Research indicates that the integration of healthcare systems, especially in rural hospitals, is essential for providing comprehensive hypertension management and reducing disparities [14]. Additionally, studies have shown that socioeconomic factors significantly influence healthcare access, which directly affects the quality of care received by minority populations [15]. For example, lower socioeconomic status is often associated with reduced access to healthcare resources, leading to poorer management of chronic conditions such as hypertension [16]. Moreover, community-based interventions, such as the use of community health workers and telehealth technologies, have been shown to mitigate some of these disparities by improving access to care in underserved populations [17].

Patient engagement is a critical determinant of healthcare quality and outcomes. Effective engagement strategies, particularly those that include community-based programs and patient education, have been shown to improve the management of chronic conditions like hypertension, especially in minority populations who often face systemic healthcare disparities. These findings are supported by research indicating that community involvement and education are key to enhancing patient outcomes, particularly in rural and underserved settings where access to healthcare is limited [14,15]. Additionally, improving health literacy through targeted patient education and community engagement has been identified as a pivotal factor in managing chronic diseases. Studies have demonstrated that health literacy can be significantly influenced by environmental factors, lifestyle choices, and local partnerships, all of which contribute to better patient outcomes [18,19].

Healthcare quality and patient outcomes

**Figure 4:**
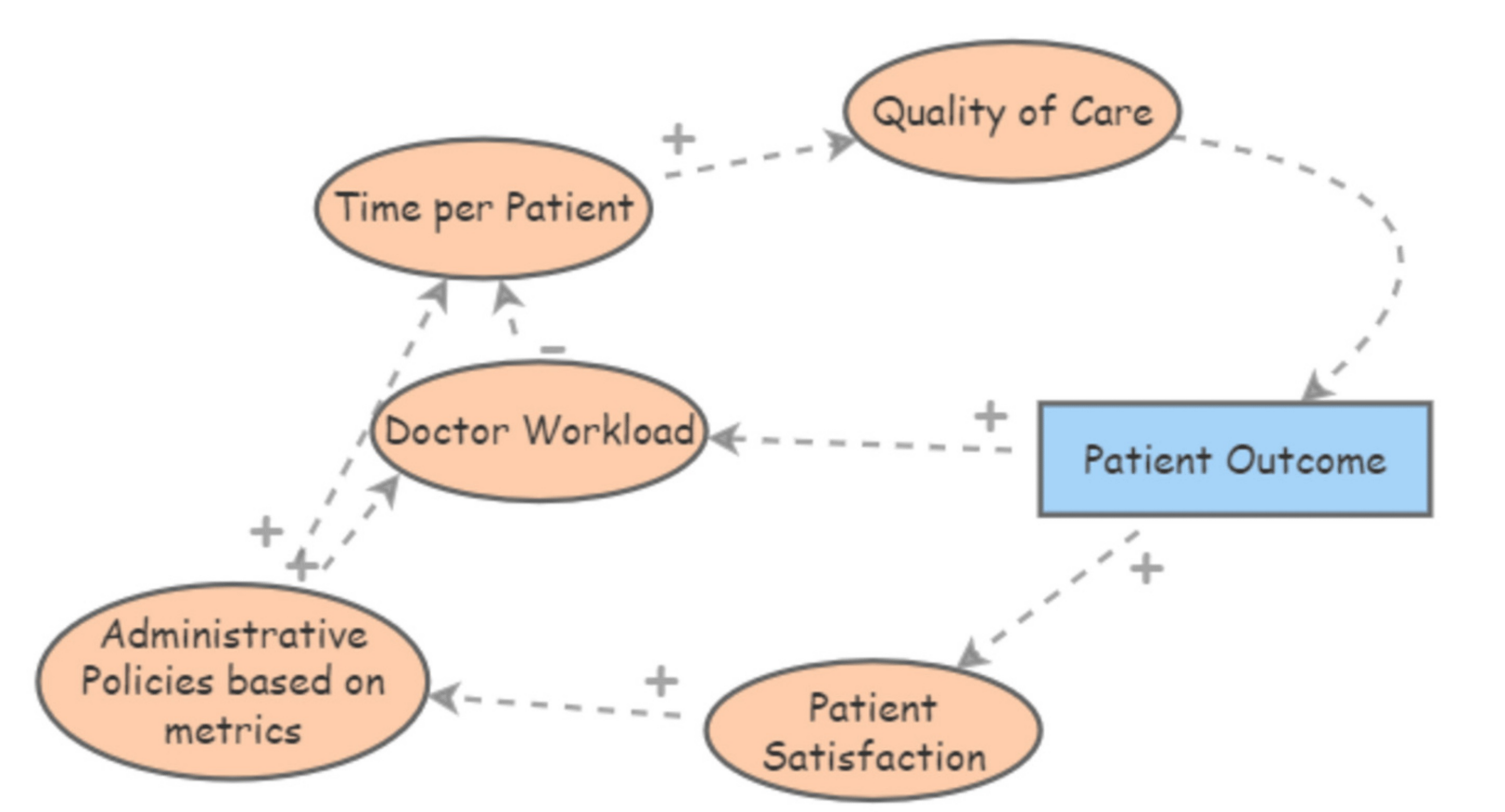
Causal loop diagram depicting the link between healthcare quality and patient outcomes

Figure 4 demonstrates the feedback loops between healthcare quality, patient engagement, and hypertension outcomes. The relationship between doctor workload and hypertension outcomes is crucial in understanding the broader impacts of healthcare management on chronic disease control. High doctor workloads, often driven by administrative policies focused on metrics, limit the time doctors can dedicate to each patient. This reduction in time negatively impacts the quality of care provided, which is particularly concerning in the management of hypertension, a condition requiring careful monitoring and patient engagement [20]. Research indicates that increased administrative tasks, including those related to electronic health records (EHRs) and population health management tools, contribute significantly to physician burnout.

This burnout is associated with lower quality of care and decreased patient satisfaction, which can directly affect hypertension management and control [20]. In particular, administrative policies that prioritize efficiency over patient interaction can exacerbate this issue, as doctors are pressured to see more patients in less time, reducing the opportunity for thorough hypertension management and follow-up [21,22]. Addressing these challenges requires a re-evaluation of administrative policies to ensure they support rather than hinder quality patient care. By reducing the technological and administrative burdens on doctors, and allowing more time per patient, healthcare systems can improve both patient satisfaction and hypertension outcomes [23,24]. This approach is critical for managing chronic conditions effectively, as it enables more personalized care and better long-term health outcomes [20].

The developed models were instrumental in identifying critical intervention points where targeted actions could most effectively address systemic issues that exacerbate health inequities. However, it is important to note that the models were built based on existing literature and secondary data, rather than direct patient interviews or primary data collection. Patient satisfaction rates, doctor workload, administrative policies, and quality of care were all inferred from established research rather than measured directly within the study. Future research should aim to include primary data collection through patient interviews, direct measurements of workload, and in-depth analysis of administrative policies to validate and expand upon these models. Despite these limitations, the models offer valuable insights into the dynamic interactions within the healthcare system and suggest evidence-based strategies for improving health equity in underserved populations.

## Discussion

This study highlights the persistent and significant disparities in hypertension control among patients at CHC, emphasizing the necessity for systemic interventions. Comparing CHC data with the NHANES data from 2011-2014 reveals several critical points.

Firstly, racial disparities in hypertension control are substantial. The CHC data show that Black/African American patients have lower hypertension control rates (61.6%) than White patients (68.6%), consistent with NHANES data reporting lower control rates in non-Hispanic Black adults (48.5%) than non-Hispanic White adults (55.7%). While it is widely understood that Black individuals are at scientifically significantly higher risk of hypertension [25], this study focuses on the disparity in achieving better healthcare outcomes in those already diagnosed. Of note, the higher baseline risk of hypertension in Black populations may exacerbate these disparities, making it doubly challenging to achieve control. Ignoring this factor could alter the statistical outcomes and limit the efficacy of interventions if they are not tailored to address this higher risk. The results stress the importance of strategies to enhance hypertension management and control in these communities. For instance, the Barbershop Hypertension Study, which provided hypertension care in Black-owned barbershops, successfully improved blood pressure control among Black men. Also, expanding community health worker programs that deliver culturally relevant education and support may further reduce these disparities [26,27].

Second, the impact of insurance status on hypertension control is significant. The CHC data reveals a stark contrast between insured and uninsured patients, with control rates of 67.7% and 37.5%, respectively. Although the sample size for uninsured patients in the CHC data is relatively small, this disparity is also reflected in NHANES data, which shows better control among insured individuals. The significant association between insurance status and hypertension control emphasizes the critical role of healthcare access in managing chronic conditions like hypertension [28,29]. Policy recommendations to improve insurance coverage include expanding Medicaid and creating state-funded insurance programs for low-income individuals [30]. Additionally, reducing barriers to obtaining insurance, such as simplifying the enrollment process and providing assistance in multiple languages, can improve coverage and outcomes. For instance, the Affordable Care Act's Medicaid expansion has been associated with improved hypertension outcomes in several states [31].

Third, age-related trends in hypertension control indicate that older adults have a higher prevalence but not proportionately higher control rates. Both CHC and NHANES data show that while hypertension prevalence increases with age, control rates do not improve proportionately. For instance, among CHC patients, control rates are similar across age groups, with no significant differences observed. NHANES data reports control rates of 57.2% for adults aged 40-59 and 52.5% for those aged 60 and over. This suggests that age-specific interventions are necessary to improve hypertension management in older populations [32,33]. Programs such as regular blood pressure monitoring and medication management tailored for older adults, as well as community exercise and wellness programs, could help improve control rates in this demographic. An example is the Systolic Blood Pressure Intervention Trial (SPRINT), which demonstrated that intensive blood pressure management in older adults significantly reduced cardiovascular events [34,35].

Fourth, systems dynamics modeling offers valuable insights into the complex interactions influencing hypertension outcomes. By visualizing the relationships between socioeconomic factors, healthcare access, and hypertension control, these models identify key leverage points for interventions. However, it is important to acknowledge that the models used in this study were based on generalized data and may not fully capture the unique challenges faced by Black populations who have a higher inherent risk of hypertension. The models suggest that interventions such as multidisciplinary care teams, community health worker programs, and telehealth services can effectively reduce disparities by addressing systemic issues [36,37]. Improving healthcare quality through evidence-based guidelines and patient-centered care can enhance patient engagement and adherence to treatment plans, leading to better hypertension control outcomes. For instance, the use of telehealth for hypertension management during the COVID-19 pandemic has shown promising results in maintaining blood pressure control [38].

Achieving health equity in hypertension management requires comprehensive, systemic interventions. Traditional approaches have proven insufficient in closing the gap in hypertension control. The study emphasizes the need for integrated interventions that address socioeconomic, healthcare, and policy-related factors [39]. Future research should focus on testing these interventions' effectiveness and exploring their long-term impacts on health equity [40]. Addressing the social determinants of health, such as income, education, and living conditions, is crucial for improving hypertension outcomes. For example, housing stability and access to nutritious food have been shown to affect blood pressure control [41,42]. Additionally, leveraging technology, such as AI-driven personalized health coaching and telehealth, can enhance patient engagement and management, particularly in underserved areas [43].

Expanding access to affordable medication is essential. Policies that increase insurance coverage for hypertension medications and provide subsidies for low-income individuals should be advocated for. Collaborating with pharmaceutical companies to create discount programs for essential hypertension drugs can also be beneficial [44]. Implementing health and wellness programs in schools, integrating these programs into school curricula, focusing on nutrition, physical activity, and stress management, and including regular blood pressure screenings for students and educational workshops for parents can promote long-term health behaviors [45]. Establishing peer support networks for individuals with hypertension, particularly in underserved communities, and training peer mentors to provide support, share experiences, and assist with managing hypertension can be impactful.

Leveraging AI-driven personalized health coaching through an app that provides personalized health coaching based on individual health data, offering tailored advice on diet, exercise, and medication adherence, and adjusting recommendations based on real-time data from wearable devices should be considered. Piloting this app in underserved communities and measuring its impact on hypertension control rates can provide valuable insights [46]. Promoting community-based participatory research (CBPR) and establishing CBPR programs where community members work alongside researchers to identify local barriers to hypertension control and co-create solutions can be effective. Implementing these solutions and evaluating their effectiveness through community feedback and health outcomes is crucial [47].

Deploying mobile clinics equipped with telehealth capabilities and integrated services such as mental health support, nutritional counseling, and hypertension management, and regularly visiting remote or underserved areas to provide on-site care and track the impact on patient outcomes and hypertension control can address gaps in healthcare access [48]. Creating workplace wellness programs by partnering with employers to include regular blood pressure screenings, stress management workshops, and incentives for healthy behaviors can help. Tracking employee participation and health outcomes to evaluate program effectiveness is important [49].

By integrating systems dynamics modeling with targeted healthcare strategies, we can design effective interventions that promote health equity and improve hypertension control outcomes [50]. However, it is crucial that future models and interventions explicitly account for the higher baseline risk of hypertension in Black populations to ensure that the strategies developed are truly effective and equitable.

## Conclusions

This study shows the critical need for systemic interventions to address the significant disparities in hypertension control among patients at CHC. Our findings highlight that racial and insurance status disparities persist despite ongoing public health efforts. The use of systems dynamics modeling has provided valuable insights into the complex interactions between socioeconomic factors, healthcare access, and hypertension outcomes. Key leverage points identified through our models include the implementation of multidisciplinary care teams, community health worker programs, and telehealth services. These interventions target systemic issues and aim to enhance health equity by addressing the root causes of health disparities.

To achieve meaningful improvements in hypertension control, it is essential to integrate socioeconomic, healthcare, and policy-related strategies. Expanding Medicaid, creating state-funded insurance programs for low-income individuals, and reducing barriers to insurance enrollment are crucial steps. Moreover, culturally tailored community-based programs and increased support from community health workers can effectively reduce racial disparities. Further research is required to test the effectiveness of these interventions and explore their long-term impacts on health equity. By adopting a comprehensive and systemic approach, we can design and implement targeted interventions that not only improve hypertension control outcomes but also promote health equity in underserved populations.
